# ARNO is recruited by the neuronal adaptor FE65 to potentiate ARF6-mediated neurite outgrowth

**DOI:** 10.1098/rsob.220071

**Published:** 2022-09-28

**Authors:** Yuqi Zhai, Wai Wa Ray Chan, Wen Li, Kwok-Fai Lau

**Affiliations:** ^1^ School of Life Sciences, Faculty of Science, The Chinese University of Hong Kong, Shatin, N.T., Hong Kong; ^2^ Research Laboratory for Biomedical Optics and Molecular Imaging, Shenzhen Institutes of Advanced Technology, Chinese Academy of Sciences, Shenzhen, People's Republic of China

**Keywords:** ADP ribosylation factor 6, amyloid *β* a4 precursor protein-binding family b member 1, ARF nucleotide-binding site opener, neurite outgrowth

## Abstract

ADP-ribosylation factor 6 (ARF6) is a small GTPase that has a variety of neuronal functions including stimulating neurite outgrowth, a crucial process for the establishment and maintenance of neural connectivity. As impaired and atrophic neurites are often observed in various brain injuries and neurological diseases, understanding the intrinsic pathways that stimulate neurite outgrowth may provide insights into developing strategies to trigger the reconnection of injured neurons. The neuronal adaptor FE65 has been shown to interact with ARF6 and potentiate ARF6-mediated neurite outgrowth. However, the precise mechanism that FE65 activates ARF6 remains unclear, as FE65 does not possess a guanine nucleotide exchange factor (GEF) domain/function. Here, we show that FE65 interacts with the ARF6 GEF, namely the ARF nucleotide-binding site opener (ARNO). Moreover, a complex consisting of ARNO, ARF6 and FE65 is detected. Notably, FE65 potentiates the stimulatory effect of ARNO on ARF6-mediated neurite outgrowth, and the effect of FE65 is abrogated by an FE65 mutation that disrupts FE65–ARNO interaction. Additionally, the intramolecular interaction for mediating the autoinhibited conformation of ARNO is attenuated by FE65. Moreover, FE65 potentiates the effects of wild-type ARNO, but not the monomeric mutant, suggesting an association between FE65 and ARNO dimerization. Collectively, we demonstrate that FE65 binds to and activates ARNO and, consequently, potentiates ARF6-mediated neurite outgrowth.

## Graphical abstract

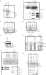

## 1. Introduction

ADP-ribosylation factor 6 (ARF6) is a small GTPase from the ARF protein family. It participates in various biological events, including vesicle transportation and cytoskeleton remodelling. There is emerging evidence demonstrating the role of ARF6 in neurite development, a process that requires rapid and dynamic cytoskeleton remodelling. Like other small GTPases, ARF6 cycles between a GTP-bound active form and a GDP-bound inactive form by the actions of guanine nucleotide exchange factors (GEFs) and GTPase-activating proteins (GAPs), respectively [1,2].

FE65, also known as amyloid precursor protein-binding family B member 1, is a 97 kDa brain-enriched adaptor protein with three distinct protein–protein interaction domains: a tryptophan–tryptophan (ww) domain and two successive phosphotyrosine-binding (ptb) domains. Using these domains, FE65 complexes with different interacting partners trigger various biological processes including the regulation of cytoskeleton dynamics [3]. We previously demonstrated that FE65 interacts with ARF6 via its PTB1 domain and potentiates ARF6-mediated neurite outgrowth [4]. As FE65 does not possess any catalytic domain/activity, it remains unknown how FE65 activates ARF6. Notably, it has been demonstrated that the activation status of ARF6 is altered by molecules that facilitate the recruitment of GEFs or GAPs. For example, ARF6 activity is modulated by PTB domain-containing engulfment adapter protein 1 through the recruitment of both ARF6 and its GAP, ACAP1 [5]. As a molecular adaptor, it is possible that FE65 stimulates ARF6 by interacting with an ARF6 regulator.

One ARF6 GEF, namely ARF nucleotide binding site opener (ARNO) which is a member of the cytohesin family of GEF proteins [6,7], is of interest as it has been shown to participate in neuritogenesis [8–10]. Of note, ARNO has been found to complex with adaptor proteins to activate signalling pathways. For example, insulin signalling is promoted by a complex consisting of ARNO and the connector enhancer of KSR1 [11]. Furthermore, ARNO interacts with interaction protein for cytohesin exchange factors 1 to facilitate epidermal growth factor signalling [12]. Similar to the PTB1 domain, the PTB2 domain of FE65 has been found to be essential for ARF6-mediated neurite extension [4]. Thus, we postulated that ARNO may be recruited by the FE65 via the PTB2 domain. Here, we report that FE65 PTB2 domain interacts with ARNO and potentiates ARF6-mediated neurite outgrowth. Moreover, FE65 attenuates the intramolecular association of ARNO to adopt an autoinhibited conformation.

## 2. Material and methods

### 2.1. Plasmids

Myc-tagged FE65 and FE65 deletion mutants were as described. Myc-tagged ARNO binding defective FE65 was generated by mutating Arg605 to Ala (R605A) using the QuikChange II site-directed mutagenesis kit (Agilent Technologies). Myc/His-tagged ARF6 construct was as described. Flag-tagged ARNO construct (p3XFLAG-2-mCytohesin-2) was a gift from Prof. Junji Yamauchi. GFP-tagged ARNO was generated by subcloning mouse full-length ARNO cDNA into the pEGFP-C1 (Clontech). The cDNA of monomeric ARNO (amino acid residues 50-399) was PCR amplified and inserted to pCMV-Tag2 vector for mammalian expression of FLAG-tagged protein. The mammalian expression vector of glutathione transferase (GST) pCIneo-GST was prepared as described [4]. Mammalian and bacterial expressing ARNO fragments were generated by inserting the corresponding cDNAs into pCIneo-GST and pGEX-6p-1, respectively. The cDNA of FE65 PTB2 (amino acid residues 531-666) was PCR amplified and inserted to pET28 for expression of the His-tagged recombinant protein.

### 2.2. Antibodies

Goat anti-FE65 (E-20), mouse anti-ARF6 (3A-1), mouse anti-ARNO (H-7), mouse anti-c-Jun (D-11) and mouse anti-α-Tubulin (DM1A) were purchased from Santa Cruz. Rabbit anti-ARNO, rabbit anti-GFP, rabbit anti-His and rabbit anti-FLAG were obtained from Proteintech. Mouse anti-myc antibody (9B11) and rabbit anti-COX IV (3E11) were obtained from Cell Signaling Technology. Mouse anti-pan-cadherin (C1821), mouse anti-β-COP (maD) and mouse anti-FLAG antibody (M2) were obtained from Sigma. Goat anti-GST antibody was obtained from GeneTex. Mouse anti-GAPDH (AM4300) was purchased from Ambion. Rabbit anti-β-Tubulin was purchased from Abcam. Rabbit anti-FE65 was as previously described [13,14]. Rat polyclonal antibodies against ARF6, ARNO and GST were created by immunization of rats with ARF6, ARNO and GST bacterial proteins, respectively.

### 2.3. Cell culture and transfection

Chinese hamster ovary (CHO), human embryonic kidney 293 (HEK293), HEK293 FE65 knockout (KO) and rat E18 primary cortical neurons were cultured as described previously [13,14]. CHO and HEK293 cells were transfected with polyethyleneimine (PEI). Primary neurons were transfected with EndoFectin Max Transfection Reagent (Genecopoeia) following the manufacturer's instructions.

### 2.4. Gene knockdown

Knockdown (KD) of specific gene expression in HEK293 cells and primary neurons were achieved by transfecting corresponding ON-TARGET plus siRNA (Horizon Discovery) with RNAiMAX (Thermo) according to manufacturer's instructions.

### 2.5. Generation of FE65 knockout cells

FE65 knockout (KO) HEK293 cells were generated as described [14]. In brief, the single guide RNAs (sgRNAs) targeting to FE65 exon 2 were designed by using an online database (http://crispr.mit.edu). The sequences of the oligos were as following: FE65 sgRNA #1_F (5′ CACCGTGTTGGCATTAATGGCCGAC 3′), FE65 sgRNA #1_R (5′ AAACGTCGGCCATTAATGCCAACAC 3′), FE65 sgRNA #2_F (5′ CACCGAAGGACCTGCGCAGCGCCAT 3′) and FE65 sgRNA #2_R (5′ AAACATGGCGCTGCGCAGGTCCTTC 3′). The designed oligos were inserted into BbsI digested pSpCas9(BB)-2A-Puro (PX459) [15]. The cells were co-transfected with the sgRNAs for 48 h and selected with 3 µg mL^−1^ puromycin (Invivogen) for 36 h. The expression level of FE65 in the selected clones was confirmed by using western blot.

### 2.6. Protein-binding assays

Mammalian GST fusion protein binding assays were performed as previously described [13,14]. In bacterial GST fusion protein pulldown assays, ‘baits’ were generated using bacterially expressed GST and GST-tagged proteins as previously described. The ‘baits’ were incubated with corresponding transfected cell lysates for protein capture. Co-immunoprecipitation assays were performed in rat brain homogenate and transfected cell lysates as previously described. Direct interaction assays were performed as described [13,14].

### 2.7. ARF6 activation assay

ARF6 activation was performed using an active ARF6 pulldown kit (Cell Biolabs) as previously described [4]. The amount of activated ARF6 in the pull downs was determined by western blotting using an anti-ARF6 antibody.

### 2.8. Neurite length measurement

Neurite length measurements were performed as previously described in a blind manner [13,14,16]. The length of the longest neurite from a transfected neuron was measured by the ImageJ (NIH) with NeuronJ plugin. At least 40 cells were analysed for each group. Statistical analyses were performed using a one-way ANOVA test with Bonferroni *post hoc* test. Differences were considered significant at *p* < 0.05.

### 2.9. Endosome isolation

Endosome was isolated from HEK293 cells by density gradient ultracentrifugation as previously described [14,17,18]. The purity of the endosome fractions was validated by probing the samples with various subcellular compartment marker antibodies.

### 2.10. Plasma membrane isolation

Plasma membrane (PM) was isolated from HEK293 cells by using a Qproteome Plasma Membrane Protein Kit (Qiagen) as previously described [13,14]. 30% of the elution was used for SDS-PAGE analysis, and 1% of total lysate was loaded as a size control. Various subcellular compartment marker antibodies were employed in western blot analyses to determine the purity of the plasma membrane preparations.

### 2.11. Immunofluorescence and colocalization analysis

The staining of ARNO, ARF6 and FE65 in HEK293 cells, and the growth cone of cultured primary cortical neurons were performed as previously described [13,14]. Images were captured using Leica TCS SP8 confocal microscope with HC PL APO CS2 63×/1.40 oil objective. Colocalization analyses were performed as previously described by using ImageJ with Coloc2 plugin, with the immunolocalization quantification strategy being Li's intensity correlation quotient (ICQ) [13,14,19]. Three independent experiments were performed in a blind manner with at least 40 cells analysed. Statistical analyses were performed using a one-way ANOVA test with Bonferroni *post hoc* test. Differences were considered significant at *p* < 0.05.

### 2.12. Proximity ligation assay

Proximity ligation assay (PLA) was performed in HEK293 cells by using a Duolink In Situ-Fluorescence kit (Sigma) as described [13,14,20]. Mouse anti-myc 9B11 and goat anti-GST or mouse anti-FLAG (M2) and goat anti-GST were used as primary antibodies for labelling the endogenous proteins. Cells were also stained with rabbit anti-β-tubulin as a morphology marker.

### 2.13. Densitometric analysis of western blots

Densitometric analysis of western blots was performed by using ChemiDoc Touch Imaging System (Bio-Rad) and analysed with Image Lab Software (Bio-Rad). Data were obtained from at least three independent experiments.

### 2.14. Statistical analysis

All experiments were repeated at least three times. Statistical analyses were performed using one-way ANOVA with Bonferroni *post hoc* test or unpaired *t*-test. Significance is indicated as *p < 0.001; **p < 0.01; ***p < 0.05; n.s., not significant (*p* > 0.05), respectively. Error bars show either s.d. or SEM.

## 3. Results

### 3.1. FE65 directly interacts with ARNO through the PTB2 domain

**Figure 1.**
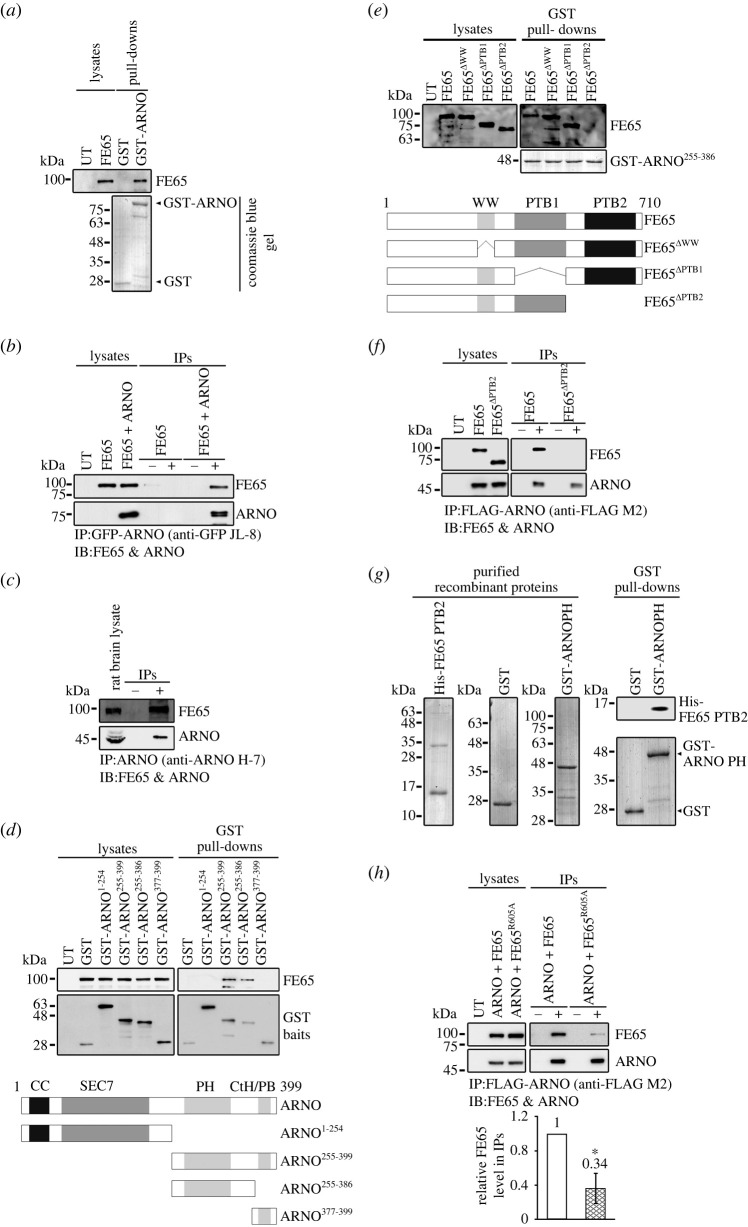
(*Overleaf*.) FE65 interacts with ARNO. (*a*) FE65 interacts with ARNO in a GST pulldown assay. Bacterially purified GST and GST-ARNO were used as baits to capture FE65 from the transfected cell lysate. FE65 in the lysates and pulldowns was detected with a goat anti-FE65 (E-20) antibody. The bait amounts in the pulldowns were revealed by a Coomassie blue gel. (*b*) FE65 interacts with ARNO in co-immunoprecipitation assays from transfected cells. Cells were transfected with either ARNO or ARNO + FE65. ARNO in the transfected lysates was captured with a mouse anti-GFP (JL-8) antibody. FE65 and ARNO in the lysates and immunoprecipitants were detected with goat anti-FE65 (E-20) and rabbit anti-GFP antibodies, respectively. ‘−’ and ‘+’ denoted the absence and presence of JL-8 antibody in the corresponding immunoprecipitation. (*c*) FE65 interacts with ARNO at the endogenous level. ARNO was immunoprecipitated from rat brain homogenate by using a mouse anti-ARNO (H-7) antibody. FE65 and ARNO in the rat brain lysate and immunoprecipitants were detected with goat anti-FE65 (E-20) and rabbit anti-ARNO antibodies, respectively. ‘–’ and ‘+’ denoted the absence and presence of H-7 antibody in the immunoprecipitations, respectively. (*d*) ARNO PH domain is required for interacting with FE65. Cells were transfected with FE65 and either GST, GST-ARNO^1-254^ (CC + Sec7), GST-ARNO^255-399^ (PH + CtH/PBR), GST-ARNO^255-386^ (PH) or GST-ARNO^377-399^ (CtH/PBR), respectively. The transfected cell lysates were incubated with glutathione Sepharose 4B for capturing the GST proteins. FE65 in the lysates and pulldowns were detected with a goat anti-FE65 (E-20) antibody, while GST proteins were detected with a rat anti-GST antibody. The bottom panel shows schematic diagrams of various GST-tagged ARNO fragments used in the assay. (*e*,*f*) FE65 PTB2 domain is needed for interacting with ARNO. (*e*) Bacterially purified GST-ARNO^255-386^ (PH) was used as bait to capture various FE65 deletion mutants from the transfected cell lysates as indicated. FE65 in the lysates and pulldowns was detected with goat anti-FE65 (E-20) antibody. The bait amounts in the pulldowns were revealed by a Coomassie blue gel. The bottom panel shows schematic diagrams of various FE65 deletion mutants used in the assay. (*f*) Cells were transfected with either FE65 + ARNO or FE65 *Δ*PTB2 + ARNO, respectively. ARNO was immunoprecipitated with a mouse anti-FLAG (M2) antibody. FE65 and ARNO in the lysates and immunoprecipitants were detected with goat anti-FE65 (E-20) and rabbit anti-ARNO antibodies, respectively. ‘−’ and ‘+’ denoted the absence and presence of M2 antibody in the immunoprecipitations, respectively. (*g*) FE65 directly interacts with ARNO. Bacterially purified GST and GST-ARNO^255-386^ (PH) were used to capture the purified His-tagged FE65 PTB2 domain. The left panel shows the Coomassie blue gel of the recombinant proteins. The right panel shows the pulldown assays. His-FE65 PTB2 in the pulldown was detected with anti-His antibody. (*h*) FE65 Arg605 is essential for interacting with ARNO. Cells were transfected with ARNO, together with either FE65 or FE65^R605A^, respectively. Mouse anti-FLAG (M2) antibody was used to immunoprecipitate ARNO from the transfected cell lysate. FE65 and ARNO in the lysates and immunoprecipitants were detected with goat anti-FE65 (E-20) and rabbit anti-ARNO antibodies, respectively. ‘−’ and ‘+’ denoted the absence and presence of M2 antibody in the immunoprecipitations, respectively. The bar chart shows the fold change of relative FE65 level in the immunoprecipitants ± s.d. Three independent experiments were performed, and statistical analysis was performed using unpaired *t*-test. *p < 0.001.

To determine whether FE65 interacts with ARNO, we first performed a bacterial glutathione S-transferase (GST) pulldown assay. *Escherichia coli*-expressed GST and GST-ARNO were used as baits to pull down FE65 in transfected cell lysates. The specific pulldown of FE65 was detected using GST-ARNO (figure 1*a*). Next, we performed an immunoprecipitation assay to confirm the FE65–ARNO interaction. ARNO was immunoprecipitated from cells transfected with FE65 and FE65 + ARNO. As shown in figure 1*b*, FE65 was co-immunoprecipitated with ARNO. The FE65–ARNO interaction was also detected in an immunoprecipitation assay of rat brain lysate (figure 1*c*), suggesting that FE65 interacts with ARNO endogenously.

Next, we determined the regions in ARNO and FE65 that are responsible for their interaction. To do this, GST-tagged constructs comprising different ARNO domains were generated (figure 1*d*, bottom panel) and used to pull down FE65 from the transfected cells. FE65 could only be pulled down by the Pleckstrin homology (PH) domain-containing constructs (i.e. GST-ARNO^255-399^ or GST-ARNO^255-386^; figure 1*d*). Deletion of the FE65 PTB2 domain (FE65*Δ*PTB2) abolished the FE65–ARNO interaction in pulldown assay using GST-ARNO^255-386^ as bait (figure 1*e*). The importance of the FE65 PTB2 domain was further confirmed by a co-immunoprecipitation assay in which FE65, but not FE65*Δ*PTB2, co-immunoprecipitated with ARNO (figure 1*f*). We also performed an *in vitro* binding assay using recombinant His-tagged FE65 PTB2 and GST-ARNO^255-386^ to determine whether FE65 directly interacts with ARNO. His-tagged FE65 PTB2 was pulled down by GST-ARNO^255-386^, but not GST (figure 1*g*). Using alanine screening mutagenesis, we found that the arginine 605 residue (R605) of FE65 was critical for the FE65–ARNO interaction, as the interaction was markedly weakened by an R605A mutation (FE65^R605A^) as determined in a co-immunoprecipitation assay (figure 1*h*). Together, our data showed that ARNO is a novel interactor of FE65 and their interaction is mediated by the FE65 PTB2 and ARNO PH domains.

### 3.2. The FE65-ARNO interaction potentiates ARF6 activation and neurite outgrowth

**Figure 2.**
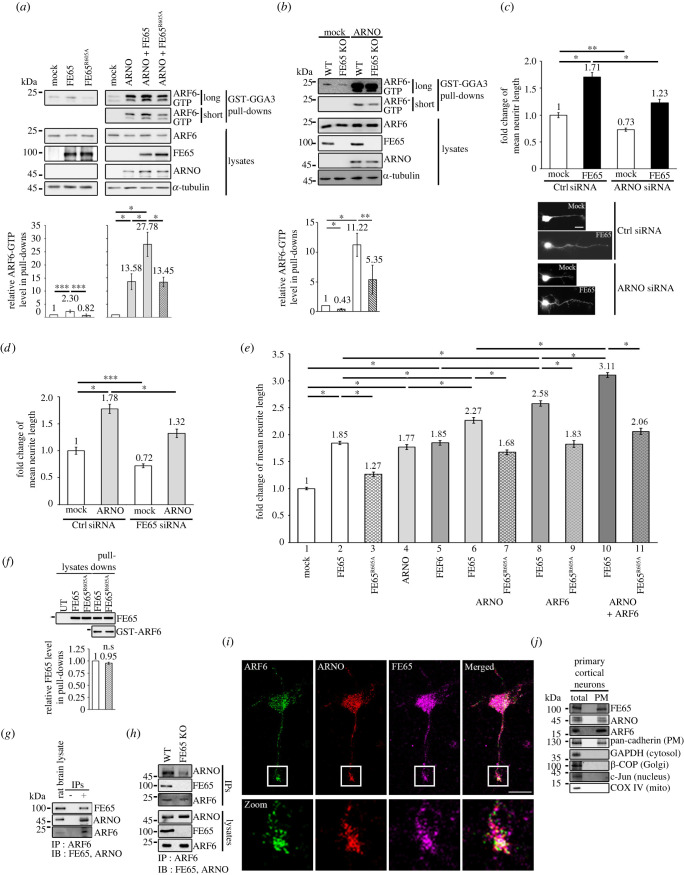
(*Overleaf*.) FE65-ARNO interaction potentiates ARF6 activation and neurite outgrowth. (*a*,*b*) FE65–ARNO interaction potentiates ARF6 activation. Cells were transfected as indicated. Activated ARF6 in cell lysates was captured by GGA3 baits. The amount of ARF6-GTP was analysed by immunoblotting. Bar charts show the fold changes of relative amount of ARF6-GTP (i.e. ARF6-GTP in pulldown/total ARF6 in lysate). Myc-tagged ARF6, FE65, ARNO and α-tubulin were detected using mouse 9B11, rabbit anti-ARNO, mouse anti-α-tubulin (DM1A) antibodies, respectively. (*a*) ARF6 activation was stimulated by FE65 overexpression but not FE65^R605A^. ARNO potentiated ARF6 activation. More potent effects were observed in co-expression of FE65 and ARNO. The expression of FE65^R605A^ inhibited such effect. (*b*) KO of FE65 reduces the effect of ARNO on ARF6 activation. (*c*–*e*) FE65–ARNO interaction potentiates neurite outgrowth. Rat embryonic cortical neurons were transfected with plasmid DNAs and/or siRNAs as indicated. EGFP was co-transfected as a morphology marker. DNAs used for transfections were of same amount. Bar charts show the fold changes of mean neurite length of the longest neurite. (*c*) KD of ARNO reduces the effect of FE65. Representative neuron images were shown. (*d*) KD of FE65 reduces the effect of ARNO. (*e*) FE65 and ARNO potentiates neurite outgrowth but not FE65^R605A^. More potent effects were observed in the neurons co-transfected with FE65 + ARNO and FE65 + ARF6. Further potentiation was observed in the FE65 + ARF6 + ARNO co-transfected neurons. Potentiation effect was not observed in the neurons co-transfected with FE65^R605A^. (*f*) FE65^R605A^ does not affect FE65–ARF6 interaction. Bacterially purified GST and GST-ARF6 were used as baits to capture FE65 and FE65^R605A^ from the transfected cell lysates. FE65 in the lysates and pulldowns was detected with a goat anti-FE65 (E-20) (1: 5000) antibody. The bait amounts in the pulldowns were revealed by a Coomassie blue gel. (*g*) FE65 interacts with ARNO and ARF6 simultaneously at endogenous level. ARF6 was immunoprecipitated from rat brain homogenate. Immunoprecipitated FE65, ARNO and ARF6 were detected using goat anti-FE65 (E20) antibody, rabbit anti-ARNO antibody and mouse anti-ARF6 3A-1 antibody, respectively. ‘−’ and ‘+’ denoted the absence and presence of mouse anti-ARF6 antibody in the immunoprecipitations, respectively. (*h*) FE65 mediates the formation of ARNO–FE65–ARF6 complex. ARF6 was immunoprecipitated from wildtype and FE65 KO HEK293 cells by using mouse anti-ARF6 (3A-1) antibody. Immunoprecipitated FE65, ARNO and ARF6 were detected using goat anti-FE65 (E20) antibody, rabbit anti-ARNO antibody and mouse anti-ARF6 (3A-1) antibody. (*i*) ARF6, ARNO and FE65 colocalize in growth cone. Rat embryonic cortical neurons were immunostained at DIV3 for the detection of endogenous ARF6, ARNO and FE65 using mouse anti-ARF6 3A-1, rat anti-ARNO, rabbit anti-FE65, respectively. Zoomed area of boxes with growth cones are shown. Scale bar is 10 µm. A representative neuron is shown. (*j*) ARF6, ARNO and FE65 localize to the PM. PM of rat primary cortical neurons was isolated using a Qproteome Plasma Membrane Protein Kit (Qiagen). Total lysate and plasma membrane (PM) elution were analysed by immunoblotting with anti-FE65 (E20), anti-ARF6 (3A-1) and anti-ARNO, respectively; together with various subcellular compartment marker antibodies including pan-cadherin, c-Jun, β-COP, COX IV and GAPDH.

As ARNO is a reported GEF for ARF6, we hypothesized that FE65 interacts with ARNO to potentiate ARF6 activation. To test this hypothesis, an ARF6 activation assay was performed. Activated ARF6 was pulled down from cells transfected with FE65, FE65^R605A^, ARNO, ARNO + FE65, and ARNO + FE65^R605A^. Overexpression of FE65, but not FE65^R065A^, stimulated ARF6 activation (figure 2*a*, left panel). Notably, co-expression of FE65 and ARNO substantially enhanced ARF6 activation. Again, FE65^R605A^ did not potentiate ARNO-mediated ARF6 activation (figure 2*a*, right panel). We also found that ARNO-mediated ARF6 activation was significantly reduced in FE65 KO cells (figure 2*b*).

As stated above, both ARNO and FE65 participate in neurite development. Therefore, we investigated their effects on neurite extension using an enhanced green fluorescence protein-based neurite outgrowth assay. As illustrated in figure 2*c*, FE65-mediated neurite outgrowth was markedly attenuated in ARNO KD neurons. Similarly, the KD of FE65 reduced the effect of ARNO on neurite outgrowth (figure 2*d*). To further delineate the role of the FE65–ARNO interaction, the binding-defective mutant FE65^R605A^ was used. Neurite outgrowth was enhanced in rat embryonic cortical neurons transfected with either FE65 (figure 2*e*, bar 1 versus 2) or ARNO (figure 2*e*, bar 1 versus 4). The stimulatory effect of FE65 was markedly attenuated by the FE65^R605A^ mutation (figure 2*e*, bar 2 versus 3). Co-transfection of ARNO with FE65, but not FE65^R605A^, further potentiated neurite extension (figure 2*e*, bar 2/4 versus 6 versus 7).

In addition to ARNO, our previous study demonstrated an interaction between ARF6 and FE65 via its PTB1 domain. The combined overexpression of FE65 and ARF6 stimulated neurite outgrowth more than the individual overexpression of FE65 or ARF6 (figure 2*e*, bar 2/5 versus 8). However, FE65^R605A^ did not potentiate ARF6-mediated neurite outgrowth (figure 2*e*, bar 8 versus 9). A GST-ARF6 pulldown assay showed that the FE65^R605A^ mutation did not interfere with the FE65–ARF6 interaction (figure 2*f*). This observation suggested that ARNO is required for FE65/ARF6-mediated neurite extension, and therefore, we postulated that these three proteins may form a functional complex. To test this hypothesis, ARF6 was immunoprecipitated from rat brain lysates. FE65 and ARNO were detected in the same precipitant (figure 2*g*). Moreover, the amount of ARNO co-immunoprecipitated with ARF6 was markedly reduced in FE65 KO cells as compared with the wild-type cells (figure 2*h*). Confocal microscopic analysis showed that proportions of FE65, ARF6, and ARNO were co-localized at the growth cone (figure 2*i*), a region with active cytoskeleton dynamics for neurite extension. Moreover, biochemical isolation revealed the presence of all three proteins in the PM (figure 2*j*), where ARF6 modulates cytoskeletal remodelling. Of note, co-expression of FE65, but not FE65^R605A^, potentiated ARF6/ARNO-mediated neurite outgrowth (figure 2*e*, bar 10 versus 11). Taken together, our data suggest that ARF6/ARNO-mediated neurite outgrowth may be potentiated by FE65, at least in part, through initiating the formation of an ARNO–FE65–ARF6 complex.

### 3.3. FE65 disrupts the intramolecular interaction of ARNO

**Figure 3.**
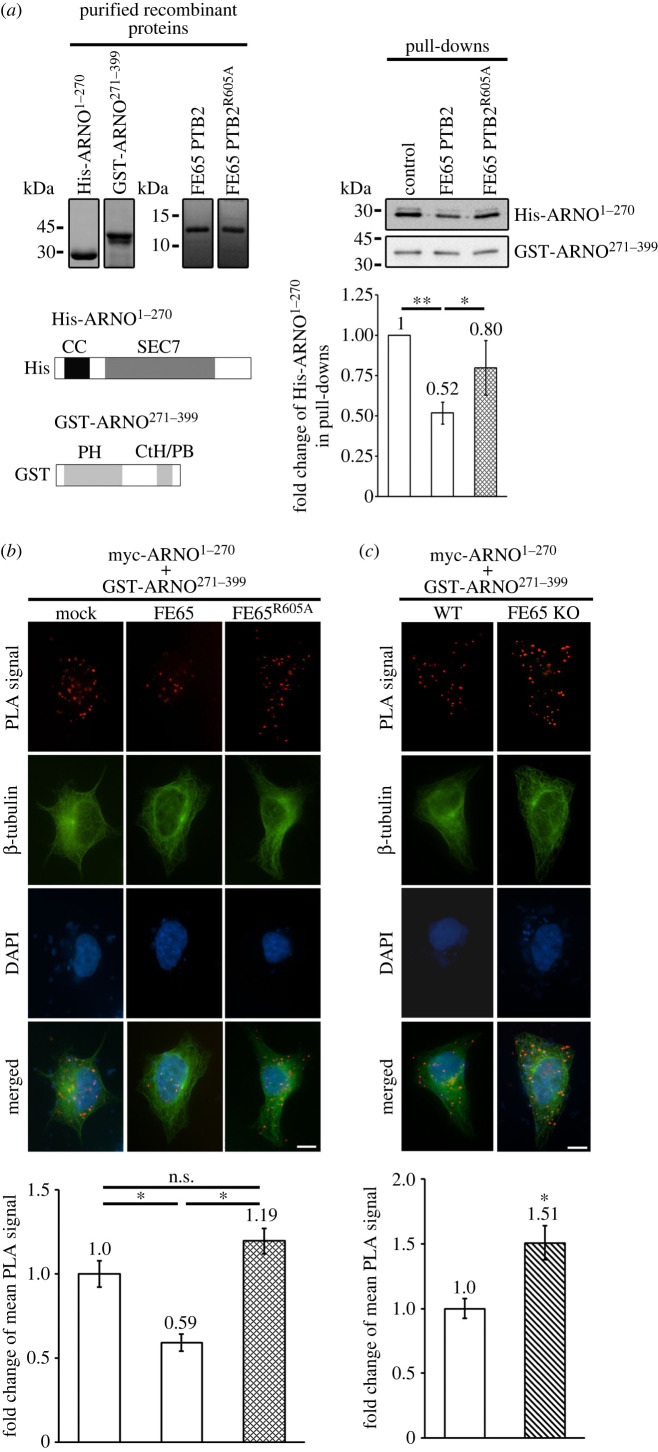
(*Overleaf*.) FE65 disrupts the intramolecular association of ARNO. (*a*) FE65 interferes ARNO fragments interaction in GST-pulldown assays. Recombinant GST-ARNO^271–399^, His-ARNO^1–270^, His-FE65 PTB2 and His-FE65 PTB2^R605A^ were expressed and purified from E coli. GST-ARNO^271–399^ was used as ‘bait’ to pull down His-ARNO^1–270^ in the presence of either His-FE65 PTB2 or His-FE65 PTB2^R605A^. The purified recombinant proteins were analysed by SDS-PAGE (left top panel). The domain structure of GST-ARNO^271–399^ and His-ARNO^1–270^ are shown (left bottom panel). The amount of His-ARNO^1–270^ in the pulldown was significantly reduced in the presence of His-FE65 PTB2, but not His-FE65 PTB2^R605A^ (right top panel). Data were obtained from three independent experiments. *, *p* < 0.001. Error bars, s.d. (*b*) FE65 interferes ARNO fragments interaction in PLAs. Myc-ARNO^1–270^ and GST-ARNO^271–399^ were transfected into HEK293 cells with mock, FE65, or FE65^R605A^. (*c*) Myc-ARNO^1–270^ and GST-ARNO^271–399^ were transfected to WT and FE65 KO HEK293 cells. In (*b*,*c*), mouse anti-myc 9B11 and goat anti-GST were used as primary antibodies for PLAs to capture myc-ARNO^1–270^ and GST-ARNO^271–399^, respectively. In B, reduced number of PLA signals were observed in the cells co-transfected with FE65 as compared with mock transfection or cells co-transfected with FE65^R605A^. More PLA signals were observed in FE65 KO cells than in the WT counterpart. In B and C, scale bar = 10 µm. Cells were stained with anti-β-tubulin and DAPI as morphology and nucleus markers, respectively. The bar charts show the relative fold change of PLA signal in different transfections. Data were obtained from at least 40 cells per transfection, and the experiments were repeated three times. *p < 0.001. Error bars, SEM.

Autoinhibition is a reported mechanism for regulating the activity of several families of GEFs. It usually involves an intramolecular interaction(s) in the GEF domain to hinder the access of the corresponding small GTPase [21]. Cytohesin family proteins, including ARNO, have been shown to adopt autoinhibited conformation. The intramolecular association of the Sec7 domain and the C-terminal helix/polybasic region (CtH/PBR) has been shown to be essential for the autoinhibited conformation [22]. Moreover, a role of the interaction between the PH and coiled-coil (CC) domains in the autoinhibited conformation has also been proposed [23]. Several GEF-interacting proteins have been reported to trigger the relief of GEF autoinhibition [13,24]. Therefore, we aimed to determine whether FE65 alters the intramolecular association of ARNO. His-ARNO^1-270^ and GST-ARNO^271-399^ fragments consisting of the CC + Sec7 + Sec7-PH linker and the PH + CtH/PBR domains, respectively (figure 3*a* left bottom panel), were expressed and purified from *E. coli* for GST-pulldown assays. As shown in figure 3*a*, GST-ARNO^271-399^ pulled down ARNO^1-270^. Of note, the interaction between the two fragments was significantly inhibited in the presence of the recombinant FE65 PTB2 domain, but not the FE65 PTB2^R605A^ mutant.

To confirm the above observation, PLAs were performed in cells transfected with myc-ARNO^1-270^ and GST-ARNO^271-399^. Fluorescent PLA signals were observed in the transfected cells, indicating the interaction between the two ARNO fragments. The number of PLA signals was reduced by approximately 40% in the cells co-transfected with FE65 but not FE65^R605A^ (figure 3*b*). Conversely, the association between the two ARNO fragments increased significantly in FE65 KO cells compared with wild-type cells (figure 3*c*). Collectively, these findings indicate that the FE65–ARNO interaction plays a role, at least in part, in relieving ARNO autoinhibition.

### 3.4. FE65 facilitates the endosomal recycling of ARNO

**Figure 4.**
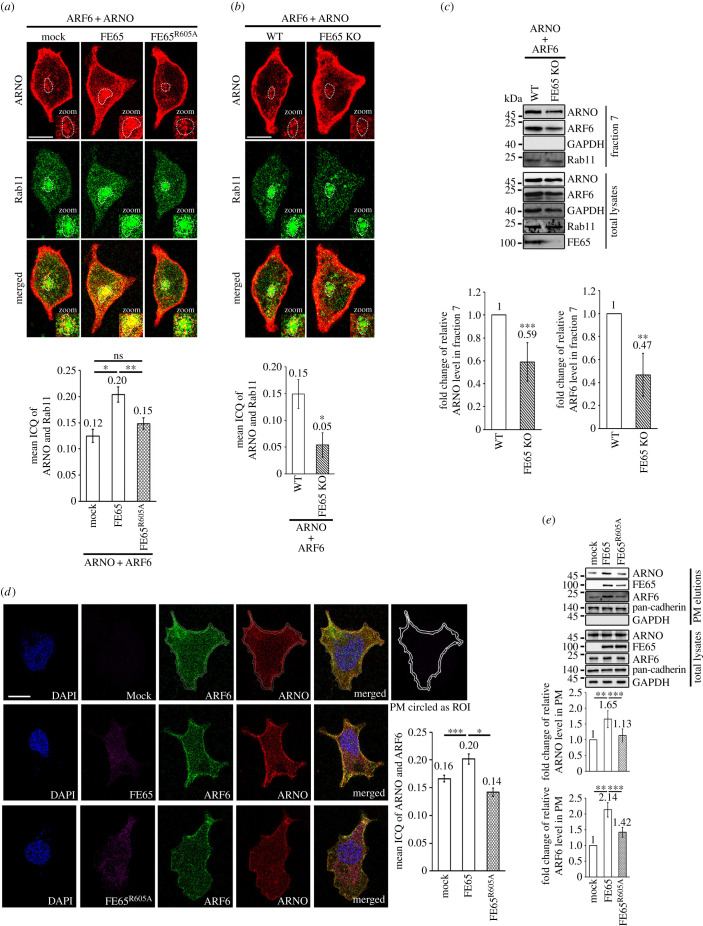
(*Overleaf*.) FE65 facilitates the endosomal recycling of ARNO. (*a*,*b*) The ERC localization of ARNO is enhanced by FE65. (*a*) HEK293 cells were transfected with either ARNO + ARF6, ARNO + ARF6 + FE65 or ARNO + ARF6 + FE65^R605A^. (*b*) WT and FE65 KO HEK293 cells were transfected with ARNO + ARF6. The cells in (*a*) and (*b*) were stained for Rab11 and ARNO by a rabbit anti-Rab11 and a mouse anti-ARNO, respectively. The co-localization of Rab11 and ARNO in the endocytic recycling compartment (ERC) (circled) was analysed by intensity correlation analyses (ICAs) using ImageJ with Coloc2 plugin. Zoomed area of boxes with ERCs are shown. For random staining, intensity ICQ = 0; for dependent staining (colocalization), 0 < ICQ < +0.5; for segregated staining, −0.5 < ICQ < 0. Scale bar is 10 µm. The intensity correlation quotients (ICQs) from at least 40 cells were obtained from each condition. Three independent experiments were performed in a blind manner. In (*a*), overexpression of FE65, but not FE65^R605A^, increased the colocalization of Rab11 and ARNO in the ERC. In (*b*) KO of FE65 significantly reduced Rab11 and ARNO colocalization in the ERC. (*c*) The amount of ARNO in the recycling endosome is reduced in FE65 KO cells. Endosomes from wildtype or FE65KO HEK293 cells were isolated by using density gradient ultracentrifugation. The protein contents in the collected fractions were analysed by immunoblotting. Rab11-positive endosome was mainly detected in fraction 7. The purity of the fractions was confirmed by analysing with various subcellular compartment marker antibodies, including Rab11, β-COP, COX-IV and GAPDH (data not shown). The amounts of ARNO and ARF6 in faction 7 were analysed. A significant reduction of ARNO and ARF6 were detected in fraction 7 from FE65 KO as compared with the wild-type counterpart. Data were obtained from three independent experiment. **p < 0.01; ***p < 0.05 Error bars, SD. (*d*) FE65 increases the co-localization of ARF6 and ARNO at the PM. HEK293 cells were transfected with either ARF6 + ARNO, FE65 + ARF6 + ARNO or FE65^R605A^ +ARF6 + ARNO. FE65, ARNO, ARF6 were stained with rabbit anti-FE65 and rat anti-ARNO and mouse anti-ARF6 (3A-1), respectively. The colocalization of ARF6 and ARNO on the PM within the region of interest (ROI) was analysed by ICA as stated in (*a*). An example of circled ROI was illustrated in the top row. Scale bar is 10 µm. Bar chart shows the mean ICQ of ARNO and ARF6. For random staining, intensity ICQ = 0; for dependent staining (colocalization), 0 < ICQ < +0.5; for segregated staining, −0.5 < ICQ < 0. Expression of FE65, but not FE65^R605A^, increased the colocalization of ARNO and ARF6 on the PM. At least 40 cells were analysed from each condition. *p < 0.001; ***p < 0.05. Error bars, SEM. (*e*) FE65 promotes ARNO and ARF6 PM distribution. PM fractions of HEK293 cells transfected with either ARF6 + ARNO, FE65 + ARF6 + ARNO, or FE65^R605A^+ARF6 + ARNO were isolated using a Qproteome Plasma Membrane Protein Kit (Qiagen). Total lysates and PM elutions were analysed by immunoblotting with anti-FE65 (E20), anti-ARF6 (3A-1) and anti-ARNO, respectively; together with marker antibodies including pan-cadherin (PM) and GAPDH (cytoplasm).

Endosomal recycling is a pathway for membrane trafficking between the recycling endosome and the PM. The PM is a compartment where ARF6 regulates the dynamics of the cytoskeleton [25]. As FE65 has been shown to modulate endosomal recycling [14], we enquired whether FE65 influences endosomal recycling of ARNO. Endocytic recycling compartment (ERC) is a tubular and vesicular membrane structure that regulates cargo recycling to the PM, and Rab11 is concentrated in this structure [26–28]. We therefore analysed the colocalization of ARNO and Rab11 in the compartment, using confocal microscopy and intensity correlation analyses. Cells were transfected with either ARNO + ARF6, ARNO + ARF6 + FE65 or ARNO + ARF6 + FE65^R605A^ and then stained for ARNO and Rab11, a marker of the recycling endosome. Significant colocalization of ARNO and Rab11 was observed in the presence of FE65 (intensity correlation quotient [ICQ], 0.20 ± 0.014 versus mock, 0.12 ± 0.012; *n* = 40; *p* < 0.001; figure 4*a*). By contrast, FE65^R605A^ showed no significant effect on the colocalization of ARNO and Rab11 (ICQ, 0.15 ± 0.011; figure 4*a*). In FE65 KO cells, a marked reduction in the colocalization of ARNO and Rab11 was detected (ICQ wild-type versus KO, 0.15 ± 0.027 versus 0.05 ± 0.023; figure 4*b*). Biochemical isolation experiments also revealed a decrease in the levels of ARNO and ARF6 in the recycling endosome-enriched fraction from FE65-KO cells (figure 4*c*). We further analysed the effect of FE65 on the colocalization of ARF6 and ARNO in the PM. We observed the colocalization of ARF6 and ARNO (ICQ 0.16 ± 0.006) on the cell surface (figure 4*d*). The colocalization of ARF6 and ARNO increased significantly when they were co-transfected with FE65 (ICQ 0.20 ± 0.006), but not FE65^R605A^ (ICQ, 0.14 ± 0.007). Moreover, the amounts of ARNO and ARF6 in PM fraction were markedly increased in the cells co-transfected with FE65, but not the mutant counterpart, as compared with the control (figure 4*e*). Hence, our data suggest that FE65 promotes the targeting of ARNO to the PM through the endosomal recycling pathway.

### 3.5. ARNO dimerization is required for FE65-mediated neurite outgrowth

**Figure 5.**
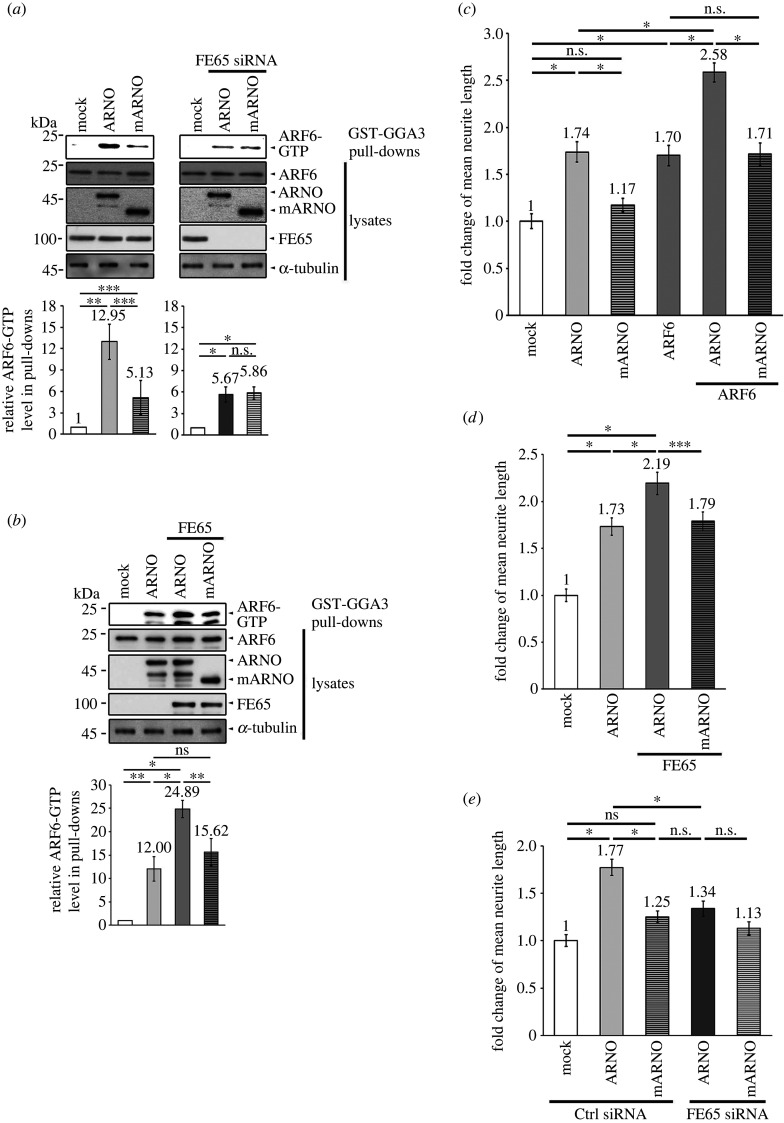
ARNO dimerization is required for FE65-mediated neurite outgrowth. (*a*) FE65 KD hinders the GEF function of ARNO but not mARNO. Cells were transfected either with mock, ARNO, mARNO, ARNO + FE65si, or mARNO + FE65si. The level of activated ARF6 in the cell lysates was analysed with an active ARF6 pulldown kit (Cell Biolabs) according to the manufacturer's instructions. ARNO was more potent in activating ARF6 than the mARNO (left panel). The effect of ARNO, but not mARNO, on ARF6 activation was reduced markedly in FE65 KD cells (right panel). (*b*) FE65 potentiates ARF6 activation in the presence of ARNO but not mARNO. Activated ARF6 in mock, ARNO, ARNO + FE65 and mARNO + FE65 transfected cell lysates were analysed. Co-expression of FE65 could only potentiate the effect of ARNO, but not mARNO, in ARF6 activation. In (*a*) and (*b*), bar charts show the mean fold change of relative ARF6-GTP level in the pulldowns ± SD. For (*a*) and (*b*), three independent experiments were performed. *p < 0.001; **p < 0.01; ***p < 0.05; n.s., *p* > 0.05. (*c*–*e*) Rat embryonic cortical neurons were cultured and transfected with plasmid DNAs and/or siRNAs as indicated for neurite outgrowth analyses. All transfections received the same amount of DNAs and/or siRNAs. (*c*) ARNO, but not mARNO, promoted neurite outgrowth either in the presence or absence of ARF6. (*d*) Co-expression of FE65 potentiated the effect of ARNO, but not mARNO, on neurite extension. (*e*). The effect of ARNO, but not mARNO, on neurite outgrowth was significantly attenuated in FE65 KD neurons. For (*c*–*e*), bar charts show the fold change of mean neurite length ± SEM. At least 40 cells were counted in each transfection. Three independent experiments were performed. *p < 0.001; **p < 0.01; ***p < 0.05; n.s., *p* > 0.05.

As stated earlier, ARF6 regulates cytoskeletal remodelling at the PM. ARNO has been shown to form homodimer via its CC domain, which facilitates its translocation to the PM [29]. To determine the role of ARNO dimerization in ARF6 activation, we used a monomeric ARNO mutant (mARNO), in which the first 49 amino acid residues were deleted [30]. We found that the ability of mARNO to activate ARF6 was greatly attenuated compared with the wild-type ARNO (figure 5*a* left panel). The effect of ARNO, but not mARNO, on ARF6 activation was markedly reduced in FE65 KD cells (figure 5*a*; wild-type cells versus FE65 KD cells, 12.95 versus 5.67 fold increase). The potencies of ARNO and mARNO in activating ARF6 were similar (figure 5*a* right panel). On the other hand, overexpression of FE65 only potentiated the effect of ARNO, but not mARNO, on ARF6 activation (figure 5*b*). Similarly, mARNO did not stimulate neurite outgrowth either in the presence or absence of ARF6 (figure 5*c*).

As we showed that FE65 promoted ARF6 activation in the presence of wild-type ARNO but not mARNO, we tested the combined effect of FE65 and ARNO or mARNO on neurite outgrowth. We found that FE65 further potentiated neurite outgrowth only in neurons co-transfected with ARNO, but not in those transfected with the monomeric mutant (figure 5*d*). In FE65 KD neurons, the effect of ARNO on neurite outgrowth was markedly attenuated (figure 5*e*). By contrast, there was no noticeable effect of mARNO on neurite extension in either control or FE65 KD neurons (figure 5*e*). Our data suggest that FE65 triggers ARF6 activation and neurite outgrowth in cells transfected with ARNO, but not the monomeric ARNO mutant.

## 4. Discussion

FE65 is a versatile adaptor protein that plays roles in many cellular processes. Increasing evidence suggests that FE65 participates in several small GTPase signalling pathways by interacting with small GTPases and/or their regulators. For instance, FE65 interacts with Dexras1, a member of the Ras superfamily of small G-proteins, to regulate the expression of glycogen synthase kinase 3*β* [31]. Moreover, FE65 has been found to regulate Rac1 by binding to the ELMO1/DOCK180 bipartite GEF of Rac1 [13]. A study also reported a direct interaction between FE65 and Rac1 [32]. Furthermore, ARF6 interacts with FE65 via the PTB1 domain to stimulate neurite outgrowth through a yet to be identified mechanism. It is noteworthy that deletion of either the PTB1 or PTB2 domain abrogates the ability of FE65 to stimulate neurite outgrowth [4]. Our biochemical analyses showed that the ARF GEF ARNO is a novel FE65 PTB2 domain interactor that stimulates neurite extension. We also identified a trimeric complex of ARNO, FE65, and ARF6. Importantly, FE65 potentiates the effects of ARNO on ARF6 activation and ARF6-mediated neurite outgrowth. Hence, FE65 may function in bringing ARNO and ARF6 in close proximity to activate ARF6.

Additionally, our data showed that FE65 may regulate ARNO functions by modulating the autoinhibited conformation of the GEF. Intramolecular inhibition is commonly found in GEFs, including cytohesins [22]. Cytohesin family proteins share a conserved domain structure comprising an N-terminal CC domain, a Sec7 catalytic domain, a PH domain and a CtH/PBR. It has been suggested that the Sec7–CtH/PBR and CC–PH associations participate in the intramolecular interaction of cytohesin, which occludes the catalytic Sec7 domain [22,23]. Mounting evidence indicates that autoinhibited conformation of GEFs can be altered by their interactors. For instance, binding of Rabaptin-5 to Rabex-5 GEF leads to the exposure of the catalytic site for the activation of Rab5 GTPase [24]. Conversely, the autoinhibited conformation of P-Rex2 GEF for Rho GTPases is strengthened by an interaction with PTEN [33]. It has been proposed that the PH domain of cytohesin 1 plays a significant role in relieving the autoinhibited conformation [30]. As we demonstrated that FE65 interacts with the PH domain of ARNO, it is possible that FE65 competes with the ARNO CC domain for the ARNO PH domain, consequently disrupting the intramolecular interaction of ARNO. Our findings are consistent with the current understanding of GEF/cytohesin regulation.

The dimerization/oligomerization of GEF proteins is another regulatory mechanism for nucleotide exchange activity. For example, the GEF activity of LARGE, a Rho GEF, is negatively regulated by its C-terminal region, which is responsible for oligomerization [34]. However, the mechanism(s) by which dimerization/oligomerization regulate(s) the catalytic function of cytohesin proteins remain(s) elusive. ARNO forms a homodimer via the N-terminal CC domain, which is proposed to be required for the efficient targeting of ARNO to the PM, where it activates ARFs [29,35]. Our cellular ARF6 activation assay data agree with this proposal, as wild-type ARNO was more potent than the monomeric ARNO mutant at activating ARF6. We also found that FE65 further potentiated the activity of ARNO, but not mARNO, toward ARF6. Hence, it is possible that FE65 functions in modulating ARNO dimerization.

The PM is the major site for ARF6 signalling [25]. A PH domain in the ARNO homodimer has been found to interact with membrane phospholipids, and this interaction is critical for ARF activation [30]. The mechanism whereby ARNO is trafficked to the PM remains unclear. We have shown here that the amount of ARNO in the recycling endosome is reduced in FE65 KO cells, which is consistent with a previous finding that FE65 promotes PM targeting through the endosomal recycling pathway [14]. FE65 may also assist the initial docking of ARNO to the PM, as FE65 has been shown to interact with several membrane-bound proteins that are implicated in neurite outgrowth, including low-density lipoprotein receptor-related protein [36–38].

It has been demonstrated that ARF6-ARNO functions upstream of ELMO1-DOCK180 to activate Rac1 [39]. Our previous finding shows that FE65 stimulates Rac1-mediated neurite outgrowth by connecting ARF6 and ELMO1-DOCK180 to form a multimeric complex [14]. Our data here suggest that ARNO can also be recruited into the complex by FE65 to potentiate the ARF6 activation. Thus, FE65 functions in mediating the formation of the multimeric complex to promote its trafficking to PM through the ARF6-ARNO-mediated endosomal recycling pathway. Once arrived, Rac1 on PM can be activated by ELMO1-DOCK180 Rac1 GEF in the complex. In addition to neurite outgrowth, the complex may function in other cellular events such as cell migration. It has been reported that FE65/FE65L1 KO mice show cortical dysplasia which is caused by defective neuroblast migration [40]. Noteworthy, ARF6-ARNO has been shown to stimulate Rac-mediated cell motility via ELMO-DOCK180 [39]. Hence, the multimeric complex may also participate in modulating cell migration. Furthermore, ARF6, ARNO and ELMO have been reported to function in calcium regulation [41]. Of note, the level of calcium transporter SERCA2 is increased in FE65/FE65L1 double KO mice which suggest a role of FE65 in calcium homeostasis [42]. On the other hand, both ARF6 signalling and FE65 have been associated with lipid metabolism through the regulation of lipid-modifying enzymes [43,44] and binding to the low-density lipoprotein receptor family proteins [3], respectively. As aberrant calcium homeostasis and lipid metabolism have been observed in Alzheimer's disease [45,46] and FE65 is implicated in the pathogenesis of the disease [3], it is worthwhile to determine the roles of the ARF6-ARNO-FE65-ELMO1-DOCK180 multimeric complex in both physiological and pathological conditions.

In addition to ARNO, FE65 PTB2 domain binds to the amyloid precursor protein (APP) intracellular domain (AICD). Of note, APP has been implicated in neurite extension. The soluble ectodomain of APP (sAPP) has been shown to stimulate neurite outgrowth [47–49]. By contrast, full-length APP has been reported to inhibit the process [50]. Thus, the exact roles of APP in neurite outgrowth remain to be defined. Notably, APP has been proposed to function as a cytosolic docking site for FE65 [51]. As we show here that the interaction between FE65 and ARNO potentiates neurite outgrowth, APP may inhibit the process by competing with ARNO for FE65. Therefore, mechanism(s) that governs the formation of FE65-APP and/or FE65–ARF6–ARNO complexes may modulate neurite extension. One possible mechanism is protein phosphorylation, a process that plays essential roles in neurite outgrowth [52]. Intriguingly, FE65 is a phosphoprotein with several reported phosphorylation residues that modulate FE65-APP interaction [53,54]. Phosphorylation of APP, ARF6 and ARNO are also reported [55–60]. Notably, neurite degeneration and aberrant protein phosphorylation are associated with Alzheimer's disease [61,62]. Hence, coordinated phosphorylation of APP, ARF6, ARNO and FE65 may function to regulate neurite outgrowth in normal and disease conditions by mediating the formation of different types of FE65-containing complexes.

Regeneration of the nervous system was once considered to be unachievable. However, increasing evidence suggests that suitable stimulation may cause injured neurons to regenerate. Neurite damage and atrophy are often observed following neuronal damage resulting from brain injuries, neurodegenerative diseases, and age-related neural degeneration. The activation of intrinsic pathways of neurite outgrowth may, therefore, trigger neurite regeneration [63]. Our finding that FE65 activates ARNO and consequently, neurite outgrowth, provides a novel target for stimulating neurite regeneration.

**Figure 6.**
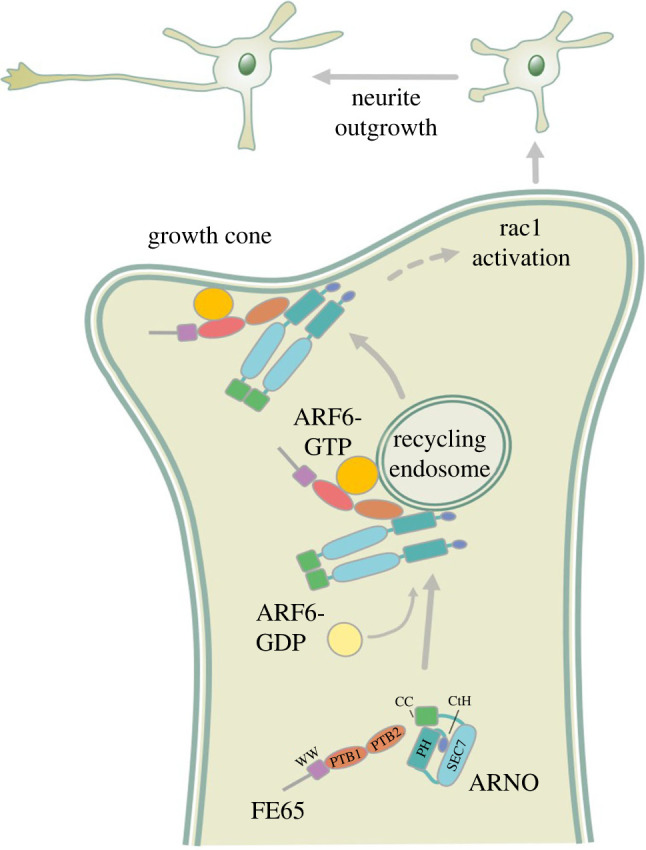
A schematic diagram illustrates the role of ARNO–FE65–ARF6 interaction in neurite outgrowth. In the growth cone, FE65 interacts with ARNO to impede the autoinhibited conformation of ARNO, consequently ARF6 activation. The FE65–ARNO–ARF6 complex is then trafficked through the endosomal recycling pathway to the PM to induce neurite outgrowth.

In summary, FE65 binds to ARNO to disrupt the intramolecular association of ARNO, thereby potentiating ARF6-mediated neurite outgrowth by targeting ARNO and ARF6 to the PM through the endosomal recycling pathway (figure 6).

## Data Availability

This article has no additional data.
